# Pro-oncogenic, intra host viral quasispecies in Diffuse large B cell lymphoma patients with occult Hepatitis B Virus infection

**DOI:** 10.1038/s41598-019-51157-1

**Published:** 2019-10-10

**Authors:** Mahua Sinha, Keerthana Sundar, C. S. Premalata, Vikas Asati, Alka Murali, Akhilesh Kumar Bajpai, Sravanthi Davuluri, Kshitish K. Acharya, K. C. Lakshmaiah, Govind Babu K., Linu A. Jacob, Dharam Nandan, Dinesh Velayutham, Sibnarayan Datta, R. S. Jayshree

**Affiliations:** 1 Department of Microbiology, Kidwai Cancer Institute, Bengaluru, India; 2 Department of Pathology, Kidwai Cancer Institute, Bengaluru, India; 3 Department of Medical Oncology, Kidwai Cancer Institute, Bengaluru, India; 4 Institute of Bioinformatics and Applied Biotechnology (IBAB), Bengaluru, India; 5 Shodhaka Life Sciences Pvt. Ltd., Bengaluru, India; 6 Agrigenome Labs Pvt Ltd, Cochin, India; 7 Molecular Virology Laboratory, Defence Research Laboratory, Tezpur, Assam India

**Keywords:** B-cell lymphoma, Tumour virus infections

## Abstract

Non Hodgkin lymphoma, predominantly Diffuse Large B-cell Lymphoma (DLBCL) has been reported to have a significant association with Hepatitis B virus (HBV). We investigated the presence of different gene segments of HBV in plasma, B-cells and tumor tissues from DLBCL patients and explored the genetic variability of HBV within and across different compartments in a host using Next Generation Sequencing. Despite all 40 patients being HBV seronegative, 68% showed evidence of occult HBV. Sequencing of these gene segments revealed inter-compartment viral variants in 26% of them, each with at least one non-synonymous mutation. Between compartments, core gene variants revealed Arg94Leu, Glu86Arg and Ser41Thr while X gene variants revealed Phe73Val, Ala44Val, Ser146Ala and Ser147Pro. In tumor compartments *per se*, several mis-sense mutations were detected, notably the classic T1762A/A1764G mutation in the basal core promoter. In addition, a virus surface antigen mis-sense mutation resulting in M125T was detected in all the samples and could account for surface antigen negativity and occult HBV status. It would be interesting to further explore if a temporal accumulation of viral variants within a favored niche, like patients’ lymphocytes, could bestow survival advantage to the virus, and if certain pro-oncogenic HBV variants could drive lymphomagenesis in DLBCL.

## Introduction

Diffuse large B cell lymphoma (DLBCL) is predominant among the Non-Hodgkin’s Lymphoma (NHL) subtypes, accounting for nearly 60% of NHLs^1^. In the last decade, serological studies have demonstrated an increased prevalence of NHL in patients infected with Hepatitis B Virus (HBV) infection^2–4^. Risk of B-cell subtypes of NHL, especially DLBCL has been documented to be significantly elevated with an odds ratio of 3.3 in HBV infected patients^2,5–8^. However, these studies were predominantly on HBV seropositive cases. Occult HBV infection (OBI), which is the presence of HBV DNA in liver and/or serum in surface antigen seronegative individuals with/without prior medical history of HBV infection^9^, may further strengthen the association of HBV with these lymphomas. Prolonged virus persistence has been suggested as an invariable outcome of HBV infection in majority even after ‘recovery’^9,10^.

Hepatotropism of HBV is well established and its association with hepatocellular carcinoma has been studied for decades^11^. However, the lymphoid system is now acknowledged as an important reservoir of HBV^6,12^. HBV lymphotropism has immense clinical implications in medical management of patients especially in a cancer centre – in providing an alternative niche for maintaining long-term virus latency, in covert viral transmission through blood transfusion or bone marrow transplantation, in clinical reactivation of hepatitis especially in patients receiving immunosuppressive chemoimmunotherapy and in promoting lymphopoietic malignancies or lymphomas among others.

Selection pressure exerted by the host immune surveillance, the virus’ error-prone replication and resulting mutations propel evolution of HBV quasispecies, which accumulate within the host and get compartmentalized in immunologically preferred sites. These viral ‘quasispecies’ are related but nonidentical viral genomes which evolve and are selected out by a continuous process of genetic variation, competition and ‘*natural selection*’^13^. In OBI, immunologically fit quasispecies are compartmentalized in diverse niche including B cells^13,14^. We explore the possibility that long-term persistence of pro-oncogenic viral quasispecies in such occultly infected B lymphocytes, probably in the presence of other pro-oncogenic co-factors, can eventually lead to B cell lymphomas. Specifically, the aim of this study was to investigate occult HBV infection in DLBCL patients and to identify viral quasispecies or variants within the host using Next Generation Sequencing (NGS).

## Results

Forty patients of histopathologically and immunohistochemically confirmed DLBCL were enrolled in a period of 1 year; 36 were newly diagnosed cases and 4 were previously diagnosed DLBCL cases on chemotherapy. The mean age of the patients was 49.22 ± 17 years (ranging from 20 to 85 years). Twenty-four (60%) patients had advanced disease (stage III and IV) while 16 patients had limited disease (stage I and stage II). Based on the Han’s algorithm there was equal distribution between molecular subtypes of DLBCL, germinal center B-cell and activated B-cell. All patients were treated with CHOP (Cyclophosphamide, doxorubicin, vincristine and prednisolone) based chemotherapy with or without anti-CD20 antibody, Rituximab. Most of the patients were from low socio-economic status and HBV specific vaccination history could not be elicited from any of our patients.

Plasma was available in all 40 cases, but B cells could be separated in 31 cases (often due to poor yield of PBMCs resulting in a futile attempt at MACS separation of B cells) and FFPE was available in only 24 cases (most often due to small lymph node tissue, not enough to spare after histopathological diagnosis). Table 1 depicts serological markers of HBV showing only 35% of the patients being seropositive to any one of the antibodies.

**Table 1:** HBV seropositivity in DLBCL.

Patient groups	Anti HBe+	Anti HBc+	Anti HBs+	Positivity for any of the 3 Antibodies
≥2 PCR positive/OBI (n = 27)	1	7	2	8/27 (~30%)
1 PCR positive (n = 9)	2	4	3	5/9 (~56%)
PCR negative (n = 4)	0	1	0	1/4 (25%)
Total (n = 40)	3 (7.5%)	12 (30%)	5 (12.5%)	14/40 (35%)

(Anti-HBe, Anti-HBc, Anti-HBs not mutually exclusive).

### HBV DNA detection

Although nearly 87.5% of patients (n = 35) had at least one HBV gene, 67.5% patients (n = 27) had OBI as characterized by PCR amplification of two or more HBV genes. Of these 27 OBI DLBCL cases, B cells were available in 24 and FFPE available in 19 patients. In these OBI DLBCL patients, HBV DNA was detectable in 85% plasma (23/27), 87.5% of B cells (21/24) and 63% of FFPE tissue samples (12/19). With respect to specific gene segments, gene C was detected in 78%, X in 74%, cccDNA (covalently closed circular DNA) and S each in 41% and P in 19% of these OBI patients. In two patients, cccDNA was detectable in the tumor tissue in addition to B cell compartment (Table 2).

**Table 2:** Patients showing HBV gene positivity across compartments.

SN	Plasma DNA +	B cell DNA +	FFPE DNA +
1	**X**, C	**X**, C, S, A	**X**
2	X, C	C, S	—
3	C	C, E	NA
4	X*, C, A	X*, P, S, E, A	—
5	X, C, A	C, S, A	—
6	C*	X, P, C*, E, A	NA
7	S*	X, S*	NA
8	C, A*	C, E, A*	NA
9	C, A	C, E	E
10	C	E	E
11	C, S, A	—	X, C
12	X	NA	X
13	S	C	S, X
14	X, C	C	NA
15	C	P, C	NA
16	—	X*, P, E, A	X*
17	X	X, E	C
18	X, C	X	C
19	X	S	X,C
20	X*	X*,S	—

Regions within X gene (X), Polymerase gene (P), Core gene (C), surface gene (S), region spanning X & precore (A), cccDNA (E). *Genes across compartments that could not be sequenced due to faint gel band, library failure, DNA poor quality or degraded. S.N. 1 - only patient where we could successfully amplify same gene across 3 compartments (**X**).

### Intra-host viral quasispecies across compartments

In OBI DLBCL, identical gene segments across at least two compartments were present in 20 patients. To identify viral variants/quasispecies, we sequenced the amplified gene sets from 20 patients using next-generation sequencing **(**Table 2**)**. After purification, quality control and library preparation, 19 sets (from 16 patients) across at least two compartments could be sequenced successfully. Variant analysis using SAMtools and BLAST showed common and unique mutations across different compartments within the host. In seven patients, distinct quasispecies were detected across compartments yielding eleven unique mutations signifying the presence of distinct intra-host quasispecies **(**Table 3**)**. Additionally, all seven patients had at least one non-synonymous mutation. The following amino acid changes could result from these mutations, with respect to the respective plasma compartment. In core gene region - G2095A (Arg94Leu) in B cell in patient 4, A2070G (Glu86Arg) in B cell in patient 31 and T1934A (Ser41Thr) in tumor cells in patient 38. In X gene region - T1590G (Phe73Val) in B cell in patients 10 and 11, C1504T (Ala44Val) in patient 36, T1809G (Ser146Ala) and T1812C (Ser147Pro) in patient 37.

**Table 3:** Patients showing intra host quasispecies across compartments.

Patient ID.	HBV Gene Region mutated	Reference position	Nucleotide detected in viral DNA from 3 host compartments			Amino acid (change) w.r.t. Ref
			Plasma	B cell	Tumor Tissue	
4	Core	2095	G	A	NA	**Arg94Leu**
10	X	1589	C	A	NA	Arg72Arg
	X	1590	T	G		**Phe73Val**
11	Core	2318	C	T	NA	Leu169Leu
	X	1590	T	G		**Phe73Val**
31	Core	2070	A	G	—	**Glu86Arg**
36	X	1504	C	T	—	**Ala44Val**
37	X	1809	T	G	—	**Ser146Ala**
	X	1812	T	C	—	**Ser147Pro**
	Precore	1888	A	G	—	Gly25Gly
38	Core	1934	T	—	A	Ser41Thr*

Amino acid abbreviations: Arginine (Arg), Leucine (Leu), Phenylalanine (Phe), Valine (Val), Glutamine (Glu), Alanine (Ala), Serine (Ser), Proline (Pro), Glycine (Gly), Threonine (Thr).

Ser41Thr* indicates a mis-sense mutant quasispecies at Core gene specifically in the tumor compartment where serine is substituted by threonine.

### Intra-compartment viral quasispecies

To understand the extent of intra host variation and mutability of the virus in our patient cohort, we analyzed abundance percentages of intra-compartment viral quasispecies in a subset of samples (nine tissue samples, 13 B cell samples and nine plasma samples). Intra-compartment quasispecies were found in majority of the samples analyzed. Four of the tumor tissue samples with 1.1 to 3.5 million reads showed over 50 to 150 quasispecies within a compartment. Majority of the viral quasispecies were found in the precore/core and X gene segments.

### Mutations in tumor compartment in X, core promoter and pre-core/core regions

We further explored sequenced data specifically from tumor tissue compartments of patients to identify significant mutations. Fifteen sequences from FFPE samples of various gene regions (seven from X, four from core, three from cccDNA, and one from S) revealed a total of 49 mutations across samples and genes. With respect to the X gene, there were 29 mutations - six in upstream region and 23 mutations in the coding region, of which 15 were mis-sense mutations resulting in amino acid change **(**Table 4**)**. The four core gene amplicons revealed 11 mutations, with two mis-sense mutations A2100C (Asp96Thr) and C2115T (Ala101Val).

**Table 4:** Mutations in HBX amplicon sequences from Tumor tissue.

SN	Mutation	Type	Domain
1	A1314G	Upstream (−60)	X promoter
2	A1320G	Upstream (−54)	
3	A1329A	Upstream (−45)	
4	T1335C	Upstream (−39)	
5	T1350C	Upstream (−24)	
6	T1353G	Upstream (−21)	
7	T1404C	Ser11Pro	Regulatory domain
8	A1437G	Ser22Gly	S/P rich region X binding region
9	C1450G	Pro26Arg	
10	G1464T	Ala31Ser	
11	A1467G	Arg32Gly	
12	G1479A	Ala36Thr	
13	G1482C	Val37Leu	
14	T1512G	Ser47Ala	
15	C1594T*	Thr74Ile	XAP1 binding Region
16	G1630A	Arg86His	
17	G1635A	Val88Ile	
18	T1762A	Met130Lys	p53 binding
19	A1764G	Ile131Val	
20	T1809G	Ser146Ala	
21	T1812C	Ser147Pro	

*Unique to patient 6. All other mutations were found in all seven FFPE samples analysed.

Amino acid abbreviations: Alanine (Ala), Arginine (Arg), Glycine (Gly), Histidine (His), Isoleucine (Ile), Leucine (Leu), Lysine (Lys) Methionine (Met), Proline (Pro), Serine (Ser), Threonine (Thr), Valine (Val).

### Mutations in S regions

Despite all 40 patients being HBsAg negative, S gene was detected in 13 patients in one or more compartments. Analysis of the S gene amplicon sequences from 10 patients identified 10 mutations - 8 mutations common to all patients (C373T, C493A, C505T, C514A, T528C, A616G, T619C, T667C), whereas 2 mutations, T364C and A541G were specific to patients 8 and 11 respectively. All the mutations were synonymous, except T528C which resulted in an amino acid change (Met125Thr) in the ‘a’ determinant of the S protein.

## Discussion

Serological studies have shown that HBV-infected patients have a higher risk of developing B-cell NHL, typically DLBCLs, than non-infected patients, and the viral infection may play an etiopathologic role in oncogenesis in these subtypes of lymphomas^2–8,15^. Our study strengthens the above virus association in a cohort of DLBCL patients by confirming presence of HBV DNA in various tissue compartments of the host despite virus surface antigen seronegativity. The study also emphasizes the presence of viral quasispecies within host tissues especially the B lymphocytes and tumor tissues that could probably propel lymphoid malignancy.

Depending on HBV endemicity, prevalence rates of OBI world-over range widely from 1–87%, being lower in healthy blood donors and higher in high-risk groups^9,16,17^. India is a region of intermediate HBV endemicity with a prevalence of OBI among healthy blood donors of 0.02 to 9% and estimated to be higher in the general population^18,19^. We found OBI in nearly 68% of the DLBCL patients.

Anti-HBc persists for years to decades following acute HBV infection and hence is often used as a surrogate marker of OBI^9^. However, OBI should ideally be diagnosed by molecular assays as anti-HBc results can be equivocal^9^ and virus replication can occur even in absence of this antibody^12,17^. Recent studies from Egypt and Japan found anti-HBc in 14% and 28.5% of DLBCLs respectively^4,15^. Likewise, we found anti-HBc in only 26% of the DLBCL patients with OBI. Overall, 35% of the patients were seropositive to any one of the viral antibodies. High prevalence of HBV DNA in DLBCL patients in the milieu of antibody seronegativity could be due to low-level viral replication not enough to stimulate immune recognition. Also, lowered affinity of the viral mutant proteins could evade diagnostic detection by commercial detection kits used^20^.

In OBI, viral replication occurs at very low levels, transiently and intermittently, making HBV DNA detection difficult^15^. Interestingly, HBV DNA was detected from not only majority of plasma and B cells but from more than half of FFPE tissue samples tested. Studies from the Middle East show an association of 24–27.5% of HBV DNA in FFPE lymph node tumor tissue from NHL patients in general (not specifically in B-cell NHL subtype)^16,21^. The relatively lower HBV DNA in tumor tissue as compared to B cell and plasma compartments in our study was expected and is attributed to FFPE DNA being highly cross-linked, degraded and fragmented. The low yield of HBV from archived tissues has been demonstrated aptly by Wang *et al*.: while B-cell NHL had HBV DNA in 55% of fresh and ~38% of archived tissue, T-cell NHL had HBV DNA in 15% of fresh and 12% of archived tissues respectively^22^.

Lymphotropism in addition to hepatotropism, is now well-recognized in hepadnaviruses including HBV^6,7,12^. Our study revealed HBV DNA in nearly 88% of B lymphocytes in the OBI DLBCL patients. Subsequent to virus infection or subclinical infection, most individuals harbour traces of HBV genome in serum and circulating lymphoid cells for years, and a replication competent HBV DNA persists in the liver or lymphocytes or both for several years or even a lifetime^6,12^. It is now known that the covalently closed circular DNA (cccDNA) of the virus that persists as an episome in the nucleus of infected/latently infected cells is the transcriptionally active viral template mainly responsible for latency. Despite the diagnostic challenge in detecting cccDNA, 40.7% of the OBI DLBCL patients were found to harbour cccDNA, suggesting replication competent virus in B cells and tumor tissue compartments of our patients. Studies on patients with past or present HBV infection have found cccDNA in 22–47% patients’ PBMCs^10,23^. Pollicino *et al*. found nearly 67% of the hepatocellular carcinoma patients to harbour cccDNA^24^.

HBV quasispecies evolve chiefly due to the virus’ error prone replication and are driven by host immune surveillance selecting out the fittest quasispecies. This continuous, dynamic process aids in virus adaptability and persistence within the host, determining clinical disease manifestation, antiviral susceptibility, emergence of vaccine escape mutants and potential viral oncogenic mutants^13^. Long term persistence of HBV causes chronic activation of B lymphocytes which may play a role in malignant transformation in lymphoid cells and tissues. The virus evolves in the B cells forming new quasispecies. Eventually the quasispecies with potential oncogenicity, in the presence of other oncogenic cofactors, probably propels lymphomagenesis, especially B-cell NHLs.

Intra-compartment viral quasispecies within host were detected in most of the samples indicating the possibility of viral variants evolving rapidly within the host and quasispecies developing temporally not only at societal level but also at individual patient level.

Inter-compartment viral variants were also detected in patients in whom the same gene segment could be sequenced across compartments. Each of these patients with viral variants had at least one mis-sense variant, either in the core gene or X gene regions, gene segments known to be implicated in oncogenesis. The HBV core protein contains two distinct domains - a putative zinc finger domain and a carboxyl terminus rich in arginine residues, indicating its DNA and RNA binding properties. The carboxy terminus domain is required for correct folding and assembly of amino terminal and central regions^25^. Mis-sense variants across compartments were localized to this domain – Arg94Leu and Glu86Arg in B cell compartment and Ser41Thr in tumor compartment. Presence of Ser41Thr specifically in tumor compartment in a patient is noteworthy as it may be a likely candidate for etiopathogenic association with DLBCL. These altered proteins amidst the virus population within the host may reflect altered nucleic acid binding and assembly of the core protein. Intra-host missense variants across compartments in X gene were located in the transactivation domain (Phe73Val), the X binding region (Ala44Val) and the p53 binding domains (Ser146Ala, Ser147Pro). Of these, Ala44Val has earlier been implicated in probable etiopathogenesis of Hepatocellular carcinoma with an odds ratio of over seven^26^ and hence may play a role in lymphomas too. The other variants can also potentially alter properties of the oncogenic X protein and may drive lymphomagenesis.

Mutations solely in the tumor compartment of patients in the X/core promoter, pre-core and core regions may also have significance in oncogenesis. Most notable of these and found in all seven tumor samples analyzed in our study was the classic double mutation T1762A/A1764G, in the basal core promoter region (BCP) which has been reported in several studies and is known to be an important pro-oncogenic mutation^26–29^. As the BCP region overlaps with the X gene reading frame, this double mutation results in amino acid changes in the X protein also (Met130Leu/Ile131Val). This mutation augments functioning of the HBX protein and in turn upregulates the expression and transcriptional activity of hypoxia-inducible factor 1-alpha (HIF-1α) contributing to tumor development and progression^30^. Furthermore, it has been shown that these mutations also promote carcinogenesis by down-regulating the expression of tumor suppressor protein, p53 via ubiquitin-mediated proteasomal degradation^29,31^. Two core promoter mutations found in all samples were the G1630A and G1635A. The G1630A mutation was also detected in another study and showed an increased association with hepatocellular carcinoma, albeit not clinically significant^26^. The G1635A mutation can be mapped to the HBV Enhancer 2 within the core promoter gene and is known to augment the enhancer and core promoter activities and subsequently HBV replication^32^. We also found double mutation T1809G/T1812C in the X gene region in all the tumor samples. This lies in the p53 binding region of the X gene and has been found to correspond with European A’e’ subgenotype. Likewise, isolates in our study showed G1809 and C1812 while African A’a’ isolates are known to have T1809 and T1812^33^. Genotype D is the predominant genotype reported from India, followed by genotypes A/C^26^. In contrast, sequencing results found most of our isolates, including the above tumor samples to be of Genotype A. Interestingly, a study from Eastern India also found Ae genotype in lymphocytes of subjects^14^. In four patients, two core mis-sense mutations, yielding Asp96Thr and Ala101Val were also from the carboxy domain of the core protein and may alter nucleic acid binding and assembly of the core protein. In addition to these mutations, several other mutations in the HBX protein were detected, significance of which are not clearly known but which may promote tumor development. Furthermore, mutations upstream of the X gene may also play a vital role in gene regulation. While the current findings indicate a possible role of such mutations in HBV mediated lymphomagenesis, further studies are needed to validate such a hypothesis.

The ‘a’ determinant within the major hydrophilic loop of the S protein contains viral epitopes which are not only targets for neutralizing antibody responses in natural infection and vaccine recipients but also the region detected in most detection assays^34,35^. Hence amino acid substitutions in this region often result in immune escapes mutants, vaccine failures and also evade detection, technically resulting in OBI^17,34,36^. We found mutant T528C (Met125Thr) within this region in all ten of our patients analyzed which may explain HBsAg negativity accounting for OBI. This mutation has also been documented in other studies from within and outside India^35,36^. Demonstration of a common M125T mutation amongst all samples sequenced resonates with the fact that point mutations are frequently observed in the first loop of the “a” epitope (a.a.124–147) of the surface antigen in OBI isolates. This could have serious public health implications: First, with regard to detection of surface antigen, such point mutations within or adjacent to the “a” determinant, alter the conformation and antigenicity of the HBV surface protein resulting in failure to detect the antigen by conventional diagnostic kits. This could imply that majority of these ‘OBI’ would go undetected in blood banks and hospitals that screen blood donors and patients solely using serology. Hence, Nucleic Acid Tests (NAT) for HBV, though not cost effective, remain the gold standard for recognition of OBI in screening blood transfusions and need to be adopted worldwide^17^. The second crucial aspect of surface gene point mutations is with regard to HBV prophylaxis. The currently used HBV vaccines are based on wild type HBS protein, and hence would fail to protect against infection by S variant viruses^17^. As a result, S variant OBI isolates circulating in the community have the potential to spread and cause break- through HBV infection even in HBV vaccinees.

Small sample size and unavailability of tumor tissue and B cells in all patients have been the major limitations of our study that explored a probable causality of HBV with this B cell lymphoma. Nonetheless, our novel findings indicate a possibility that HBV quasispecies may be spontaneously evolving and accumulating within various tissue compartments of DLBCL patients. In occult HBV infected subjects, considering the established oncogenicity of the virus and the pro-oncogenic virus mutations found in our study, one cannot rule out the possibility of a direct causative link of some such virus-variants in the development of these B cell lymphomas. While virus *in-situ hybridization* (ISH) studies would have helped in cellular localization of the signals within the tumor tissue, PCR is relatively more sensitive in virus detection. Also, targeting different virus gene fragments ensured that a sufficiently high specificity of the PCR assay was maintained. Hence, for validating the results of the present study, meticulously planned experiments in animal models, although challenging, would certainly be useful.

Overall, the study emphasizes a need for awareness about OBI and its probable insidious role in development of not only hepatomas but also B-cell lymphomas. The results of the study also underscore the need for active research into developing alternative diagnostic assay kits and better candidate vaccines encompassing HBS variants which would further a step towards containing the silent OBI epidemic in the community. Considering the large burden of B cell Non-Hodgkin lymphomas and DLBCL, a preventive healthcare strategy could certainly curtail the incidence of HBV mediated DLBCL. Although communicable diseases are generally prioritized by public health experts, there is a need to implement necessary steps to reduce the burden of these apparently “non-communicable” cancers.

## Methods

This prospective study included patients attending a tertiary care cancer centre in Southern India from January to December 2016. Before initiating the study, ethical approval was obtained by the institutional ethical committee (Kidwai Memorial Institute of Oncology, Bangalore, India, certificate dated 11^th^ March 2015). A written, informed consent was obtained from all patients before enrolment into the study. All experimental procedures were performed in accordance with relevant guidelines and regulations.

### Study subjects

Confirmed cases of DLBCL (newly diagnosed/on therapy) over 18 years of age were recruited for the study. Diagnosis was made preferably by excisional biopsy of lymph node or Tru-Cut biopsy of involved organ. In addition to routine histopathological examination, immunohistochemical markers like CD20, CD10, CD3, Bcl-2, Bcl-6, cMyc, MUM1, CyclinD1 and PAX5 were also used to confirm the diagnosis of DLBCL. Staging was done as per the standard Ann Arbor system.

Peripheral blood samples of patients were centrifuged and plasma was separated for serological and molecular tests. Peripheral blood mononuclear cells (PBMCs) were separated from remaining blood sample by density gradient centrifugation using Ficoll Histopaque (Cat. No. 17-1440-02, GE Healthcare Life sciences, Pittsburgh, USA). These mononuclear cells were washed thoroughly with phosphate buffered saline (pH 7.4) and B cells were separated by positive selection using Magnetic Assisted Cell Sorter (MACS) (CD20 Microbeads, Miltenyi, Biotec, GmbH, Germany Cat No. 130-091-104). Tumor tissue was sourced from Formalin Fixed Paraffin Embedded (FFPE) lymph node tissue. Fresh lymph node tissue, ideal for viral DNA extraction, was tried initially but eventually abandoned due to logistics of screening and storing cells of large number of suspected lymphoma patients (very often being diagnosed as NHL other than DLBCL). Also, often times, the lymph node tissue was too small to spare before making paraffin blocks for histopathological diagnosis.

### Serological markers

Hepatitis B surface antigen (HBsAg) and antibodies to HBV surface antigen, HIV and HCV were tested in all patients by standard chemiluminescent immunoassay (ARCHITECT Abbott Laboratories, Wiesbaden, Germany). Commercially available Enzyme-Linked Immunosorbent Assay-based kits were used for the detection of anti-HBc, HBeAg and anti-HBe (Diapro, Italy). All assays were carried out and results interpreted according to the manufacturer’s instructions.

### DNA extraction and PCR

DNA was extracted separately from samples, collected from three distinct compartments, using commercially available kits: (i) from approximately 10^5^ circulating B cells (QIAamp® DNA blood mini kit, QIAGEN GmbH, Hilden, Germany, Cat No. 51104), (ii) 200 µl of plasma (QIAamp® MinElute virus Spin kit, QIAGEN, Cat No. 57704) and (iii) two 10 µ sections of FFPE tumor tissue samples (black Prep®, Analytik Jena kit, Germany, Cat No. 845-BP-0020050). Minor modifications were incorporated in the extraction procedures to increase viral genome yield. For DNA extraction from B cells & FFPE tumor tissues, carrier RNA (QIAGEN, Cat No.1068337) was added in the initial lysis step each time. Additionally, for DNA extraction from FFPE tissues, proteinase K lysis step was extended to overnight digestion. In all three extractions, elution volumes were reduced to 50 μl to increase the concentration of viral DNA. Standard precautions were strictly followed for sample handling and manipulation including use of aerosol resistant tips, laminar flow work bench and unidirectional work flow in physically separated pre, per and post PCR areas, to avoid cross contamination and procedural false positivity.

### HBV nested PCR

HBV DNA was detected by nested PCR assays as per published protocol, targeting parts of the core (C)^24^, polymerase (P)^24^, surface gene region encoding the major hydrophilic loop (S)^14^, X gene with a small overlap with pre-core/core region (X)^37^ and a large region spanning the X and pre-core/core regions^38^. All PCR experiments were done in 50 µl reaction mix containing 200 µM dNTPs, 1.25U Taq DNA polymerase (New England BioLabs Inc, NEB, Cat No. N0446S & M0273L) and 0.2 µM each of forward and reverse primers. The thermal cycling profile based on NEB recommendations and our primer characteristics included incubation for 1 min at 95 °C, 30 cycles of 95 °C for 30 s, 50 °C for 1 min, and 68 °C for 1 min, followed by a final extension step at 68 °C for 5 min. Appropriate positive, negative and no template controls were included in each run to rule out possible contamination. PCR amplification products were electrophoresed on 1.5% agarose gel, stained by ethidium bromide and visualized under UV illumination.

Presence of covalently closed circular DNA (cccDNA), the intranuclear replicative template of the virus responsible for maintaining HBV latency, was discerned from relaxed circular DNA (rcDNA) genome of HBV based on sensitivity of the latter to digestion with Mung bean nuclease, following a previously described method^24^. Briefly, 5 μl of DNA extracted from FFPE tissue and B cell compartment was digested with 10U of Mung bean nuclease in 10 μl of reaction buffer (Cat No. M0250S New England BioLabs Inc) for 30 mins at 37 °C. Subsequently, reaction was stopped using 2.5 μl ethylene glycol bis NNN’N’ tetra acetic acid (EGTA). Five microlitres of the digested DNA was then used as a template in PCR.

Cases which yielded positive samples for two or more different viral gene segments or one gene segment across two or more compartments were considered as HBV DNA positive as per criteria for diagnosing OBI^24,39^.

### Next Generation Sequencing of amplicons and variant analysis

The HBV gene amplicons were sequenced (Agrigenome Labs, Cochin, India) using next generation sequencing (NGS) technology for determination of quasispecies. Fifty-six amplicons, generated with specific primers for the gene of interest were gel extracted using PureLink TM Gel Extraction kit (ThermoFisher Scientific, Carlsbad, CA, USA). Samples were verified for QC on Qubit and agarose gel followed by fluorometric analysis and the samples meeting the required QC parameters were undertaken for library preparation. Library was prepared using NEB Next DNA Ultra Lib prep kit (New England Biolabs). Quantity and quality of prepared libraries were measured using Qubit Fluorometer and Agilent 2200 TapeStation (Agilent Technologies, USA) respectively. Good quality libraries were subjected to sequencing on Illumina HiSeq. 2500 platform. The raw sequence QC was performed with FastQC^40^, adapter sequences in raw reads were trimmed using Cutadapt^41^, and the trimmed reads were aligned to the reference HBV genome (NCBI accession: KC875257.1) using BWA-mem. Mutations were identified using SAMtools^42^. Filters were applied for mapping quality (30) and read depth (1000).

The mutations resulted through the NGS data analysis pipeline were further verified using BLAST. The filtered reads were used to generate the consensus sequences for each sample, which were then matched with HBV14A genotype (KC875257) along with three additional reference genotypes of HBV viz., HBV78C (KC875274), HBV34D (KC875340), HBVAWI (NC_003977), using BLASTn with default parameters^43^. Among the four genotypes considered, HBV14A was found to be the best matched reference, and hence considered for variant analysis.
